# Clinical efficacy and safety of dotinurad, a novel selective urate reabsorption inhibitor, in Japanese hyperuricemic patients with or without gout: randomized, multicenter, double-blind, placebo-controlled, parallel-group, confirmatory phase 2 study

**DOI:** 10.1007/s10157-019-01818-2

**Published:** 2019-12-03

**Authors:** Tatsuo Hosoya, Takafumi Sano, Tomomitsu Sasaki, Masahiko Fushimi, Tetsuo Ohashi

**Affiliations:** 1 Jikei University School of Medicine, 3-25-8, Nishi-Shimbashi, Minato-ku, Tokyo, 105-8461 Japan; 2 Development Department, Medical R&D Division, Fuji Yakuhin Co., Ltd, 4-383, Sakuragi-cho, Omiya-ku, Saitama-shi, Saitama 330-9508 Japan

**Keywords:** Hyperuricemia, Gout, Selective urate reabsorption inhibitor, URAT1 inhibitor, Dotinurad, FYU-981

## Abstract

**Background:**

Dotinurad, a novel selective urate reabsorption inhibitor (SURI), reduces serum uric acid levels by selectively inhibiting urate transporter 1 (URAT1) for the treatment of hyperuricemia with or without gout. We confirmed the serum uric acid lowering effect and safety of dotinurad.

**Methods:**

This was a confirmatory, 12-week, randomized, multicenter, double-blind, placebo-controlled, parallel-group, dose escalation, late phase 2 study. The study arms were dotinurad 0.5, 1, 2, or 4 mg and placebo. The primary endpoint was the percent change in serum uric acid level from the baseline to the final visit. The secondary endpoint was the percentage of patients achieving a serum uric acid level ≤ 6.0 mg/dL at the final visit.

**Results:**

The study drugs were administered to 200 Japanese hyperuricemic patients with or without gout. The mean percent change in serum uric acid level from the baseline to the final visit in the dotinurad 0.5, 1, 2, and 4 mg groups and the placebo group was 21.81%, 33.77%, 42.66%, 61.09%, and − 2.83%, respectively. The percentage of patients achieving a serum uric acid level ≤ 6.0 mg/dL at the final visit in each group was 23.1%, 65.9%, 74.4%, 100%, and none, respectively. Regarding safety, the incidence of adverse events did not increase with dose escalation in the dotinurad groups. No significant differences were observed in the incidence of gouty arthritis in each group.

**Conclusion:**

The serum uric acid lowering effect and safety of dotinurad were confirmed in hyperuricemic patients with or without gout.

**ClinicalTrials.gov Identifier:**

NCT02416167

## Introduction

Gouty arthritis is a form of inflammatory arthritis that results from chronic hyperuricemia [1]. In recent years, hyperuricemia has been known to associate with chronic kidney disease (CKD), hypertension, and diabetes mellitus [2–4]. Furthermore, it has been reported that increased serum uric acid levels may also play a pivotal role in the progression of CKD and the development of new hypertension [5, 6]. Treatment of hyperuricemia is, thus important for the prevention of these diseases. Japanese guidelines for the management of hyperuricemia and gout recommend pharmacological therapy for hyperuricemia without gout or gouty tophi (asymptomatic hyperuricemia), especially, in cases where the serum uric acid level is ≥ 8.0 mg/dL together with lifestyle diseases such as CKD, hypertension, and diabetes mellitus [1]. In accordance with the Japanese guidelines, the management goal is to reduce and maintain the serum uric acid level at ≤ 6.0 mg/dL, which hopefully leads to the dissolution of urate crystals in the joints [1].

Hyperuricemia can be classified mainly as excessive uric acid production “overproduction type”, decreased uric acid excretion “underexcretion type”, or both conditions “combined type”. In Japan, prevalence of each is estimated to be 10%, 60%, and 30%, respectively. The basic principle of the Japanese management guidelines is to use xanthine oxidase inhibitors (XOIs) (e.g., allopurinol and febuxostat) for “overproduction type” and uricosuric drugs (e.g., probenecid and benzbromarone) for “underexcretion type” [7]. Recently, the Japanese management guidelines have been updated for the third edition, and the classification of hyperuricemia was changed from “overproduction type” to “renal load type”, because it has been understood that the conventional “overproduction type” includes the “extrarenal underexcretion type” (decreased uric acid excretion from the intestine) [1]. In the present study, the classification of hyperuricemia was implemented according to the Japanese management guidelines, second edition [7], the latest version at the start of the study.

Increased urinary uric acid excretion by uricosuric drugs fears of urinary calculi formation, and renal impairment [1]. In addition, these drugs often have a lesser serum uric acid lowering effect in patients with moderate to severe renal dysfunction [1]. For these reasons, hyperuricemic patients with renal impairment are mainly prescribed XOIs. However, allopurinol sometimes induces severe adverse drug reactions (ADRs) such as Stevens–Johnson syndrome, toxic epidermal necrolysis, and hypersensitivity syndrome of allopurinol in patients with renal impairment. These events may be due to increased levels of serum allopurinol and its active metabolite, oxypurinol, which are mainly excreted by the kidneys. Therefore, in hyperuricemic patients with renal impairment, the allopurinol dosage should be regulated [8, 9]. Furthermore, with new XOIs, such as febuxostat and topiroxostat, hepatic impairment has been observed as an ADR [10, 11].

For these reasons, there are safety concerns with the XOIs and uricosuric drugs currently in use, thus the development of safer drugs with sufficient serum uric acid lowering effect is anticipated for the treatment in majority of hyperuricemic patients.

Dotinurad is a novel selective urate reabsorption inhibitor (SURI) for the treatment of hyperuricemia in patients with or without gout [12]. In the early phase 2 study in Japanese patients, we observed a substantial serum uric acid lowering effect in patients with hyperuricemia. Regarding safety, no significant differences were observed in the incidence of ADRs between the dotinurad groups and the placebo group [NCT02344862].

We conducted this confirmatory phase 2 study to confirm the dose dependency of serum uric acid reduction, optimum dose, and safety of dotinurad in Japanese hyperuricemic patients with or without gout, including elderly patients and those with renal dysfunction.

## Methods

### Study design

This was a late phase 2, 12-week, randomized, multicenter, double-blind, placebo-controlled, parallel-group, dose escalation study performed at 14 clinical institutions in Japan.

### Inclusion and exclusion criteria

The inclusion criteria for this study were a serum uric acid level during the run-in period ≥ 7.0 mg/dL (patients with a history of gouty arthritis or gouty tophus), ≥ 8.0 mg/dL (patients with asymptomatic hyperuricemia who are receiving medication or had a diagnosis of hypertension, diabetes mellitus, and/or the metabolic syndrome), or ≥ 9.0 mg/dL (asymptomatic hyperuricemia without aforementioned complications), in Japanese patients aged 20 years or older on the day that written informed consent for participation in this study was obtained. The serum uric acid level criteria were followed by the Japanese guidelines [7].

The exclusion criteria were as follows: gouty arthritis that had not became asymptomatic within the two weeks before the day of randomization; possible disorders causing secondary hyperuricemia; hemoglobin A1c (HbA1c, NGSP) ≥ 8.4%; use of drugs that might have affected the outcome of this study during the two weeks before the starting day of the run-in period to randomization; hyperuricemia classified as indeterminate or “overproduction type”; complications of any serious cardiac disorder, a history of myocardial infarction, and/or an angina attack within a year; complications or a history of cancer (in the five years before obtaining informed consent); complications of serious hepatic impairment, aspartate aminotransferase (AST), and/or alanine aminotransferase (ALT) ≥ 100 U/L; complications of a renal calculus or clinical manifestations suspicious of a urinary calculus (e.g., hematuria, back pain); estimated glomerular filtration rate (eGFR) < 30 mL/min/1.73 m^2^; blood pressure ≥ 180 mmHg systolic and/or ≥ 110 mmHg diastolic; a history of drug allergy; and presence of any other clinically significant medical conditions that could potentially preclude participation in this study. If patients had been treated with any antihyperuricemic drug, or drugs affecting the serum uric acid level before the enrolment of this study, they were allowed to enter into this study only after a washout period of 2–4 weeks.

### Treatment

**Fig. 1:**
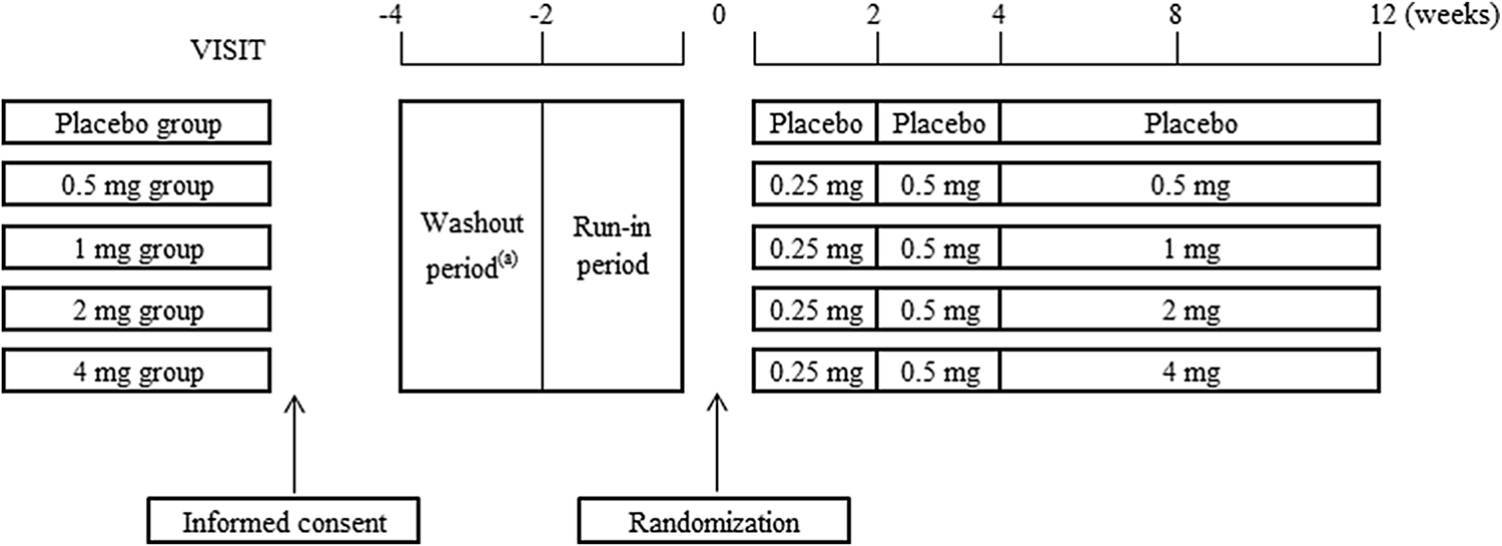
Dosing schedule. **a** Patients who had been treated with antihyperuricemic drugs or treatment affecting the serum uric acid level were subjected to the washout period

Figure 1 shows the dosing protocol of study drug. Before starting any study-related procedures, written informed consent was obtained from all the participants. At the end of the run-in period, they were randomly assigned to the dotinurad 0.5, 1, 2, or 4 mg groups or the placebo group (ratio 1:1:1:1:1). An independent organization conducted a randomized block allocation of the study drug. Patients received study drugs once daily after breakfast. To minimize the risk of gouty arthritis due to rapid serum uric acid reduction, we adopted the dose titration method [13]. The initial dose of dotinurad was 0.25 mg/day for the first 2 weeks and then 0.5 mg/day for another 2 weeks. The maintenance dose of dotinurad (0.5, 1, 2, or 4 mg/day) was provided from week 4 to the final visit. The minimum maintenance dose of dotinurad was set to 0.5 mg/day in this study, because based on the early phase 2 study, some degree of serum uric acid lowering effect could be expected even at a dose of 1 mg or less. The investigators instructed the patients to restrict excessive exercise, diet, and to drink enough water during the study.

Furthermore, to minimize the risk of a urinary calculus in association with increased urinary uric acid excretion, a urinary alkalization drug (citrate) was given together with the study drug in the following cases: (1) history of urolithiasis, (2) urine pH < 6.0 (from informed consent to end of this study), and (3) needs for the therapy at an investigator’s discretion.

To maintain the double-blind condition, the serum uric acid level was not disclosed to patients, study investigators, and local sponsor personnel from the study drug administration until the final database was disclosed.

### Classification of hyperuricemia

Based on measurement of uric acid in the 60 min urine collection during the run-in period, hyperuricemia was classified into four types: (1) uric acid overproduction type—urinary extraction of uric acid (E_UA_) > 0.51 mg/kg/h and uric acid clearance (C_UA_) ≥ 7.3 mL/min/1.73 m^2^; (2) uric acid underexcretion type—E_UA_ < 0.48 or C_UA_ < 7.3; (3) combined type—E_UA_ > 0.51 and C_UA_ < 7.3; and (4) normal type—0.48 ≤ E_UA_ ≤ 0.51 and C_UA_ ≥ 7.3. The patients classified as “uric acid overproduction type” were excluded from this study, for fear of the urinary calculus formation.

### Efficacy endpoints

The primary efficacy endpoint was the percent change in serum uric acid level from the baseline to the final visit. In addition, the secondary efficacy endpoints were the percentage of patients achieving a serum uric acid level ≤ 6.0 mg/dL at the final visit and the serum uric acid levels at each time point.

### Safety evaluations

Adverse events (AEs) and safety assessments were conducted by clinical investigators based on vital signs, 12-lead electrocardiography, clinical laboratory tests, and clinical examination throughout this study. AEs were classified according to the system organ class and preferred term (MedDRA version 19.0; Japanese Maintenance Organization, Tokyo, Japan) and were evaluated in terms of their possible causal relationship with the study drug, as well as severity and seriousness. AEs judged to be related to the study drug were defined as ADRs.

### Statistical analyses

The primary endpoint was analyzed using the Jonckheere–Terpstra test to examine dose dependency. We calculated that 20 patients were required per group to determine dose dependency, as found in the early phase 2 study. However, taking into consideration the number of patients that might be excluded from the analyses, and the number of patients who could be evaluated for safety, we set the group size to 40 patients in each group.

Efficacy was evaluated using the full analysis set (FAS), which comprised all randomized patients who received at least one dose of the study drug and underwent serum uric acid measurement during at least one visit.

If the serum uric acid level was missing at the last visit (week 12), this omission was compensated by the Last Observation Carried Forward (LOCF) method. This approach was pre-specified before the start of this study. In the efficacy analyses of the primary endpoint, the mean values between individual groups were compared using the Tukey–Kramer test and the dose dependency of dotinurad groups was examined using the Jonckheere–Terpstra test. The Cochran–Armitage test was used to evaluate the dose dependency of the secondary efficacy endpoint of the dotinurad groups. In addition, the *χ*^2^ test was used to compare the mean values between individual groups, to analyze the secondary efficacy endpoint.

Safety analyses were performed on the safety population (SP), which comprised all patients who received at least one dose of the study drug. The incidence of AEs was summarized as the number and percentage of patients. The Cochran–Armitage test was used to evaluate the dose dependency of dotinurad groups and the *χ*^2^ test was used to compare incidences between individual groups.

The statistical analyses of efficacy and safety were performed using SAS software, version 9.2 (SAS Institute Inc., Cary, NC, USA). Unless otherwise specified, all values are expressed as mean ± standard deviation (SD). Statistical significance was defined based on a two-tailed *P* value of < 0.05. Moreover, the statistical significance of between-group differences in the baseline characteristics of patients was defined based on a two-tailed *P* value of < 0.15.

## Results

### Patient flowcharts and baseline characteristics

**Fig. 2:**
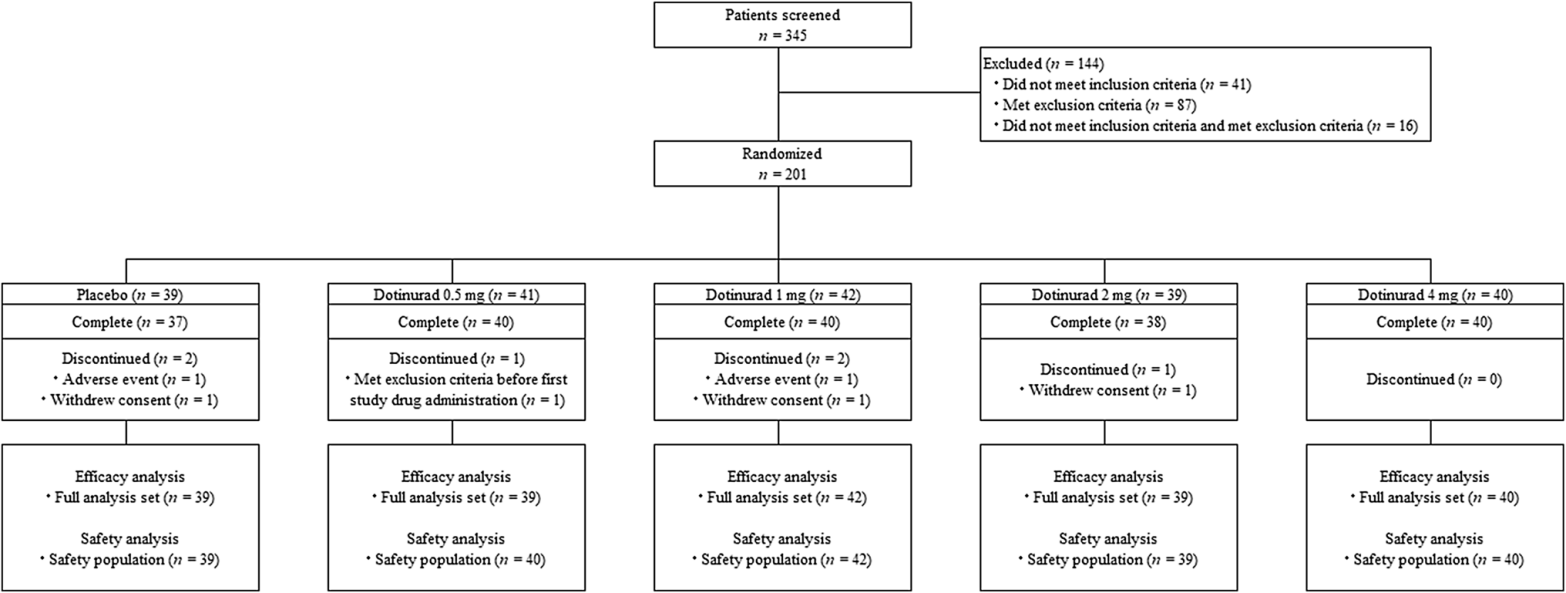
Flow diagram of study protocol

Figure 2 is a diagram of the study protocol. Within the period of May 2015 to March 2016, 345 patients were screened, 144 were excluded, and the remaining 201 were randomized to dotinurad groups (0.5 mg, *n* = 41; 1 mg, *n* = 42; 2 mg, *n* = 39; 4 mg, *n* = 40) or placebo group (*n* = 39). One patient (dotinurad 0.5 mg group) was removed for meeting the exclusion criteria before the first study drug administration. Five patients did not complete the study (1 mg, one discontinued due to AE and one withdrew consent and discontinued due to AE; 2 mg, one withdrew consent; placebo, one discontinued due to AE, and one withdrew consent and discontinued due to AE). One patient (dotinurad 0.5 mg group) who received the study drug was excluded from the FAS for meeting the exclusion criteria after study drug administration. All patients who received the allocated drug at least once were included in the SP.

The baseline characteristics of patients were comparable among all groups (Table 1). The mean serum uric acid level and mean eGFR during the run-in period ranged from 8.84 to 9.02 mg/dL and 69.2 to 71.4 mL/min/1.73 m^2^, respectively, in the overall groups.

**Table 1:** Baseline characteristics of patients enrolled

Characteristic		Placebo	Dotinurad				*P* value^a^
		(*n* = 39)	0.5 mg (*n* = 39)	1 mg (*n* = 42)	2 mg (*n* = 39)	4 mg (*n* = 40)	
Sex	Male	38	39	42	38	39	0.719^c^
	Female	1	0	0	1	1	
Age (year)	Mean ± SD	52.8 ± 11.0	58.5 ± 12.1	57.4 ± 12.4	55.0 ± 13.5	58.0 ± 10.5	0.186^b^
Height (cm)	Mean ± SD	169.86 ± 5.82	169.61 ± 6.22	167.96 ± 5.72	169.18 ± 7.33	170.64 ± 6.55	0.412^b^
Weight (kg)	Mean ± SD	76.49 ± 13.41	76.33 ± 14.93	74.26 ± 8.11	76.42 ± 17.10	79.97 ± 12.52	0.437^b^
Serum uric acid (mg/dL)	Mean ± SD	8.93 ± 1.04	9.02 ± 1.20	8.84 ± 1.07	8.96 ± 1.14	8.94 ± 1.10	0.967^b^
eGFR (mL/min/1.73 m^2^)	Mean ± SD	71.4 ± 13.9	69.2 ± 15.1	71.2 ± 15.5	71.0 ± 15.4	69.8 ± 13.9	0.953^b^
Medical history of hyperuricemia	Number of patients (%)	30 (76.9)	32 (82.1)	28 (66.7)	28 (71.8)	29 (72.5)	0.593^c^
History of gouty arthritis	Number of patients (%)	35 (89.7)	31 (79.5)	35 (83.3)	33 (84.6)	30 (75.0)	0.501^c^
Existence of gouty tophus	Number of patients (%)	1 (2.6)	0 (0.0)	0 (0.0)	2 (5.1)	0 (0.0)	0.232^c^
Drinking habits	Number of patients (%)	25 (64.1)	24 (61.5)	27 (64.3)	26 (66.7)	24 (60.0)	0.977^c^
Classification of hyperuricemia	Uric acid underexcretion type (%)	33 (84.6)	31 (79.5)	39 (92.9)	29 (74.4)	34 (85.0)	0.234^c^
	Combined type or normal type (%)	6 (15.4)	8 (20.5)	3 (7.1)	10 (25.6)	6 (15.0)	

eGFR for male (mL/min/1.73 m^2^) = 194 × Serum creatinine^−1.094^ × Age^−0.287^

eGFR for female (mL/min/1.73 m^2^) = 194 × Serum creatinine^−1.094^ × Age^−0.287^ × 0.739 [18]

Definition of drinking habit: consumption of alcohol more than 3 days of the week and consumption of more than 500 mL of beer or 60 mL of whisky in a day

^a^*P* < 0.15

^b^Kruskal–Wallis test

^c^*χ*^2^ test

### Efficacy

#### The primary efficacy endpoint

The percent changes (mean ± SD) in serum uric acid level from the baseline to the final visit in each group were 21.81 ± 11.35%, 33.77% ± 9.82%, 42.66% ± 13.16%, 61.09% ± 8.75%, and − 2.83% ± 8.19%, in the dotinurad 0.5, 1, 2, and 4 mg groups and the placebo group, respectively (Table 2), indicating that dose dependency was observed in the dotinurad 0.5, 1, 2, and 4 mg groups (*P* < 0.001, Jonckheere–Terpstra test). Furthermore, a significant difference in percent change was observed between each dotinurad group and the placebo group (*P* < 0.001, Tukey–Kramer test).

**Table 2:** Primary and secondary efficacy endpoints

End point	Category	Placebo	Dotinurad				
		(*n* = 39)	0.5 mg (*n* = 39)	1 mg (*n* = 41^a^)	2 mg (*n* = 39)	4 mg (*n* = 40)	
Percent change in serum uric acid level from the baseline to the final visit	Mean ± SD (%)	− 2.83 ± 8.19	21.81 ± 11.35	33.77 ± 9.82	42.66 ± 13.16	61.09 ± 8.75	
	95% confidence interval	− 5.49 to − 0.18	18.13 to 25.48	30.67 to 36.87	38.40 to 46.93	58.29 to 63.89	
	Jonckheere–Terpstra test	–	*P* < 0.001*				
	Tukey–Kramer test	–	*P* < 0.001*	*P* < 0.001*	*P* < 0.001*	*P* < 0.001*	
Percentage of patients with serum uric acid level ≤ 6.0 mg/dL at the final visit	Number (%)	0 (0.0)	9 (23.1)	27 (65.9)	29 (74.4)	40 (100.0)	
	95% confidence interval	0.0 to 9.0	11.1 to 39.3	49.4 to 79.9	57.9 to 87.0	91.2 to 100.0	
	Cochran–Armitage test	–	*P* < 0.001*				
	*χ*^2^ test	–	*P* = 0.001*	*P* < 0.001*	*P* < 0.001*	*P* < 0.001*	

Jonckheere–Terpstra test and Cochran–Armitage test were conducted in the groups of dotinurad 0.5, 1, 2, and 4 mg

Tukey–Kramer test and *χ*^2^ test were adjusted about placebo vs each dotinurad group

^*^P < 0.05

^a^One patient within FAS was not contained this analysis because serum uric acid level at the final visit was missed

#### The secondary efficacy endpoint

The percentages of patients achieving a serum uric acid level ≤ 6.0 mg/dL at the final visit in each group were 23.1% (9/39 patients), 65.9% (27/41 patients), 74.4% (29/39 patients), 100% (40/40 patients), and none (0/39 patients) in the dotinurad 0.5, 1, 2, and 4 mg groups and the placebo group, respectively (Table 2), indicating dose dependency in the dotinurad groups (*P* < 0.001, Cochran–Armitage test). Furthermore, significant differences were observed between each dotinurad and placebo group (*P* ≤ 0.001, *χ*^2^ test).

**Fig. 3:**
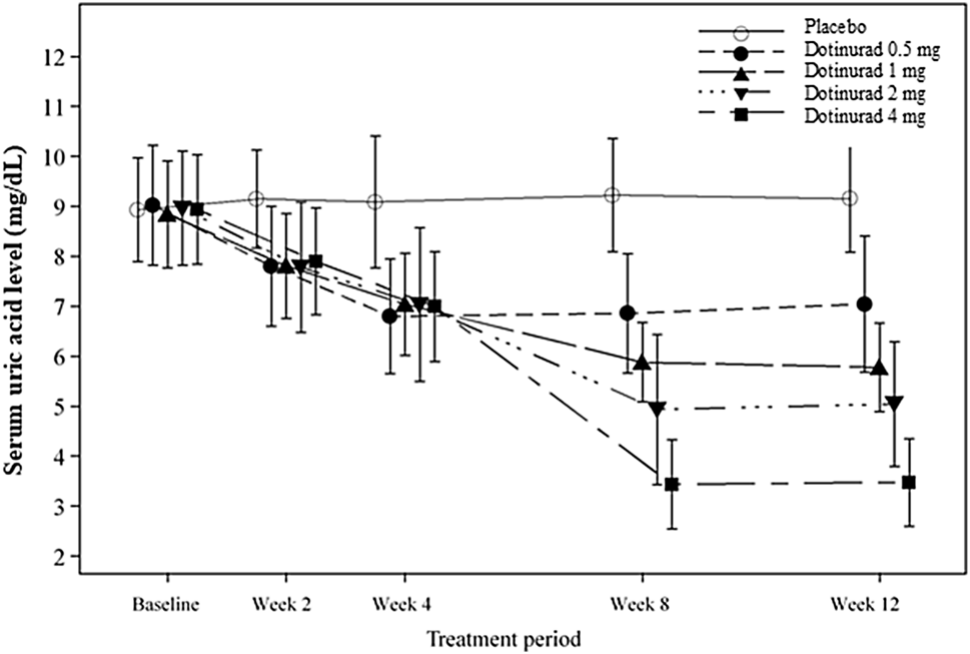
Changes in serum uric acid level in response to follow treatment with dotinurad. Error bar indicates standard deviation

Figure 3 shows the changes in serum uric acid level in response to follow treatment with dotinurad. In the dotinurad groups, the serum uric acid lowering effect was observed compared to baseline and the effect tended to be enhanced dose increased. The mean serum uric acid level at the final visit for the dotinurad 0.5, 1, 2, and 4 mg groups and the placebo group was 7.04, 5.87, 5.14, 3.48, and 9.16 mg/dL, respectively.

### Safety

Table 3 shows the incidences of AEs and ADRs in this study. AEs were observed in 24 patients (60.0%), 21 patients (50.0%), 20 patients (51.3%), 13 patients (32.5%), and 20 patients (51.3%) in the dotinurad 0.5, 1, 2, and 4 mg groups and the placebo group, respectively. A dose-dependent decrease was noted in the incidence of AEs (*P* = 0.021, Cochran–Armitage test). AEs was not associated with dose escalation; however, significant differences were observed between the 0.5 and 4 mg groups (*P* = 0.014, *χ*^2^ test). All AEs were mild or moderate in severity at an investigator’s discretion. Serious AEs were observed in three patients (colon cancer, hemorrhagic diverticulum intestinal, prostate cancer). Colon cancer and hemorrhagic diverticulum intestinal hemorrhagic occurred in the dotinurad 1 mg group and prostate cancer occurred in the dotinurad 2 mg group. A causal relationship was ruled out between all serious AEs and the study drug. AEs that caused study discontinuation, excluding serious AEs, were observed in the following: one patient in the dotinurad 1 mg group (gouty arthritis); one patient in the dotinurad 2 mg group (sciatica); and two patients in the placebo group (urinary β2-microglobulin increased, gastroenteritis). The investigators considered gouty arthritis and urinary β2-microglobulin increased to be ADRs.

**Table 3:** Incidence of AEs and ADRs

	Placebo		Dotinurad							
	(*n* = 39)		0.5 mg (*n* = 40)		1 mg (*n* = 42)		2 mg (*n* = 39)		4 mg (*n* = 40)	
	AEs	ADRs	AEs	ADRs	AEs	ADRs	AEs	ADRs	AEs	ADRs
Number of events	31	8	42	7	41	16	43	12	30	14
Number of patients	20	6	24	6	21	8	20	7	13	7
Incidence (%)	51.3	15.4	60.0	15.0	50.0	19.0	51.3	17.9	32.5	17.5
*χ*^2^ test	–		P vs 0.5 mg		P vs 1 mg		P vs 2 mg		P vs 4 mg	
*P* value (AEs)	–		0.435		0.908		1.000		0.091	
*P* value (ADRs)	–		0.962		0.663		0.761		0.800	

Incidence (%) = number of patients/number of analyzed patients × 100

*P* placebo

Regarding ADRs, the incidences in each group were comparable and no significant differences were detected among each group (*P* > 0.05, *χ*^2^ test). The incidences of ADRs did not increase with dose escalation (*P* = 0.814, Cochran–Armitage test).

Table 4 shows the incidence of gouty arthritis. Gouty arthritis was not reported in the placebo group. No significant differences were observed among each group (*P* > 0.05, *χ*^2^ test). The investigators considered all gouty arthritis events to be ADRs of mild or moderate severity.

**Table 4:** Incidence of gouty arthritis

	Placebo	Dotinurad			
	(*n* = 39)	0.5 mg (*n* = 40)	1 mg (*n* = 42)	2 mg (*n* = 39)	4 mg (*n* = 40)
Number of events	0	1	5	4	4
Number of patients	0	1	2	3	3
Incidence (%)	0.0	2.5	4.8	7.7	7.5
*χ*^2^ test	–	P vs 0.5 mg	P vs 1 mg	P vs 2 mg	P vs 4 mg
*P* value	–	0.320	0.168	0.077	0.081

Incidence (%) = number of patients/number of analyzed patients × 100

*P* placebo

## Discussion

The percent change in serum uric acid level from the baseline to the final visit was significantly higher in all dotinurad groups than in the placebo group and dose dependency was observed in the dotinurad groups. In addition, significant differences in the percentage of patients achieving a serum uric acid level ≤ 6.0 mg/dL were noted between all dotinurad groups and the placebo group.

Regarding safety, no significant differences were observed in the incidences of AEs between all dotinurad groups and the placebo group and an increased tendency toward dose dependency was not observed in the dotinurad groups. No significant differences were observed in the incidence of gouty arthritis among all groups.

In Japan, benzbromarone is recommended for treatment of “underexcretion type” patients. However, administering benzbromarone is contraindicated in patients with hepatic impairment, because serious hepatic impairment, including fulminant hepatitis, has been reported [14]. Regarding AEs related to hepatic impairment in our study, AST and ALT increases were observed in 3.1% in the dotinurad groups. All of these events were mild in severity and only one of each was judged to be an ADR by the investigators. In the placebo group, no AEs related to hepatic impairment were observed.

In recent years, although lesinurad, classified as an SURI, was approved in the United States and the European countries, renal impairment was reported as an ADR in a clinical study [15]. The renal impairment observed with lesinurad may be a result of increased urinary uric acid excretion inducing urate microcrystallization in the renal tubules [16]. However, in our study, no AEs related to renal impairment such as acute kidney injury or serum creatinine increase were observed in dotinurad, which is also classified as an SURI. Furthermore, no serious renal impairment has been reported as an ADR with other uricosuric drugs. Therefore, renal impairment with lesinurad has not been considered a class effect of uricosuric drugs.

Lesinurad, bucolome, and probenecid reportedly have attenuated serum uric acid lowering effects in patient with renal dysfunction [1, 17]. In contrast, in the subgroup analysis of this study that examined renal function at baseline, the serum uric acid lowering effect of dotinurad in patients with moderate renal dysfunction (eGFR ≥ 30 to < 60 mL/min/1.73 m^2^) was comparable those with mild dysfunction (eGFR ≥ 60 to < 90 mL/min/1.73 m^2^) and normal function (eGFR ≥ 90 mL/min/1.73 m^2^) (Table 5). Moreover, no significant safety problems were observed in patients with renal dysfunction. These results indicated that efficacy and safety of dotinurad were comparable with normal renal function or moderate renal dysfunction, suggesting that there is no need to adjust the dose based on renal function.

**Table 5:** Percent change in serum uric acid level from the baseline to final visit by the category of eGFR at the baseline

	Placebo			Dotinurad											
	(*n* = 39)			0.5 mg (*n* = 39)			1 mg (*n* = 41)			2 mg (*n* = 39)			4 mg (*n* = 40)		
eGFR category^a^	Normal	Mild	Moderate	Normal	Mild	Moderate	Normal	Mild	Moderate	Normal	Mild	Moderate	Normal	Mild	Moderate
	(*n* = 5)	(*n* = 26)	(*n* = 8)	(*n* = 4)	(*n* = 25)	(*n* = 10)	(*n* = 5)	(*n* = 27)	(*n* = 9)	(*n* = 4)	(*n* = 26)	(*n* = 9)	(*n* = 3)	(*n* = 27)	(*n* = 10)
Mean^b^ (%)	3.05	− 4.64	− 0.64	24.62	22.47	19.01	25.51	35.57	32.98	27.68	46.69	37.68	69.68	59.68	62.30
SD	7.39	8.23	7.02	6.94	9.88	15.94	17.20	8.56	6.47	7.74	11.23	14.59	0.98	9.43	6.52
95% CI	− 6.13 to 12.23	− 7.96 to − 1.32	− 6.51 to 5.23	13.57 to 35.67	18.39 to 26.55	7.61 to 30.41	4.16 to 46.87	32.18 to 38.96	28.00 to 37.96	15.37 to 39.99	42.16 to 51.23	26.47 to 48.89	67.24 to 72.13	55.95 to 63.41	57.64 to 66.97

eGFR for male (mL/min/1.73 m^2^) = 194 × Serum creatinine^−1.094^ × Age^−0.287^

eGFR for female (mL/min/1.73 m^2^) = 194 × Serum creatinine^−1.094^ × Age^−0.287^ × 0.739 [18]

^a^eGFR category: normal, eGFR ≥ 90 mL/min/1.73 m^2^; mild, eGFR ≥ 60 to < 90 mL/min/1.73 m^2^; moderate, eGFR ≥ 30 to < 60 mL/min/1.73 m^2^

^b^Mean: mean of percent change in serum uric acid level from the baseline to the final visit

Although most of participants were male in the present study, we consider dotinurad is also useful for female patients, because we confirmed that dotinurad had no clinically meaningful effect on the pharmacokinetics, pharmacodynamics by gender in the phase 1 study of dotinurad for healthy adult male and female subjects [NCT02344875].

In this confirmatory phase 2 study, the efficacy and safety of dotinurad were confirmed in hyperuricemic patients with or without gout. However, we believe that some comparison studies with existing antihyperuricemics, such as benzbromarone or febuxostat, are necessary to establish the clinical utility of dotinurad in the treatment of hyperuricemia with or without gout.

In conclusion, the efficacy and safety of dotinurad were confirmed in hyperuricemic patients with or without gout.
